# Risk Factors for Excessive Screen Exposure in Children With Epilepsy: A Cross-Sectional Study in Assiut, Egypt

**DOI:** 10.7759/cureus.85020

**Published:** 2025-05-29

**Authors:** Amira Elhoufey

**Affiliations:** 1 Department of Community Health Nursing, Alddrab University College, Jazan University, Jazan, SAU

**Keywords:** children, epilepsy, family, screen exposure, screen time

## Abstract

Purpose: This study aimed to evaluate screen exposure patterns and identify modifiable risk factors in children with epilepsy to inform targeted interventions for this vulnerable population.

Methods: A cross-sectional study was conducted at two tertiary hospitals in Assiut, Egypt, between January and December 2024. A convenience sample of 200 children aged between three and 12 years with epilepsy was recruited. Caregivers completed structured questionnaires assessing screen time, device types, and parental regulation behaviors. Univariate analyses compared screen exposure durations, while multivariate logistic regression identified predictors of excessive screen use (>1 hour/day on school days or >2 hours/day on weekends).

Results: Children aged between three and six years had significantly higher screen time than those aged between six and 12 years (mean daily: 98 ± 25 minutes vs. 87 ± 21 minutes, p < 0.001). Male children exceeded screen limits more frequently than female children (p < 0.01). Our results revealed that pre-sleep screen use (adjusted odds ratio (AOR): 3.41; 95% CI: 1.89-6.16; p < 0.001) and low parental education level (AOR: 3.39; 95% CI: 1.98-5.82; p < 0.001) remained the strongest independent predictors of excessive screen use. Additionally, caregiver discussion of screen content was found to be a significant factor, with a lack of discussion increasing the likelihood of excessive screen use by over threefold (AOR: 3.11; 95% CI: 1.44-6.73; p = 0.004). Seizure duration >5 minutes also independently predicted excessive screen exposure (AOR: 3.07; 95% CI: 1.62-5.81; p = 0.001). Notably, variables such as family income, device ownership, and total seizure count did not reach statistical significance (p > 0.05).

Conclusions: The findings of this study underscore that excessive screen exposure in pediatric patients with epilepsy is independently associated with modifiable sociobehavioral and clinical factors, including pre-sleep screen use, caregiver engagement in screen content discussions, and parental educational attainment. Interventions focused on these factors may help reduce seizure triggers and enhance overall health outcomes.

## Introduction

Screen exposure, defined as engagement in screen-based activities such as television viewing, tablet use, computer interaction, and smartphone utilization, has emerged as a significant public health concern in pediatric populations. The current research on neurotypical children generally underscores the increasing prevalence of excessive screen time, driven by technological advancements and widespread access to digital smart devices, with documented associations with adverse developmental, behavioral, and metabolic outcomes [1-2].

Children with epilepsy represent a vulnerable subgroup requiring targeted investigations. This population exhibits unique clinical and psychosocial characteristics that increase the potential risks of prolonged screen exposure. Epilepsy, ranked as the fifth most burdensome neurological disorder worldwide, accounts for over 13 million disability-adjusted life years (0.5% of the global disease burden). Notably, approximately 10% of individuals with epilepsy demonstrate photosensitivity, a markedly higher prevalence compared to less than 0.5% in the general population [3]. The multisensory stimuli inherent to screen devices, including luminance variations, flickering patterns, and chromatic contrasts, may trigger reflex epileptic seizures in photosensitive patients, rendering excessive screen exposure particularly hazardous for this cohort [4]. Concurrently, disease-specific factors contribute to increased screen dependency. Children with epilepsy often experience reduced physical activity levels due to seizure-related restrictions and parental safety concerns [5]. Furthermore, both patients and caregivers frequently report heightened social stigma, which may drive compensatory reliance on digital platforms for entertainment, socialization, and emotional regulation. International studies indicate that children with epilepsy exhibit longer screen durations than healthy peers, with inverse correlations between screen time and health-related quality of life metrics [5].

The previous data on screen exposure patterns among Egyptian children with epilepsy remain very limited.

This study aimed to evaluate screen exposure patterns and identify modifiable risk factors in children with epilepsy to inform targeted interventions for this vulnerable population. Specifically, the study aimed to determine the prevalence of excessive screen time among children with epilepsy, identify sociodemographic, clinical, and behavioral predictors of excessive screen use, and explore caregiver regulation practices associated with screen exposure.

The primary hypothesis was that pre-sleep screen use and lower parental education level would be independently associated with higher odds of excessive screen time. Additional research questions included whether seizure duration and caregiver engagement in screen content discussions would significantly influence screen exposure patterns.

The findings will inform culturally responsive interventions to optimize screen time management, mitigate seizure triggers, and enhance holistic care for children with epilepsy.

## Materials and methods

Ethical approval and consent statement

The study was approved by the Ethical Committee of Al-Azhar University Hospitals, Assiut, Egypt (Institutional Review Board (IRB) approval number: 25/172/3/2023). Informed written consent was obtained from all participating caregivers. All patient information was handled confidentially, and data were used solely for research purposes.

Study design and period

This cross-sectional study was conducted between January and December 2024 at two tertiary hospitals in Assiut, Egypt. These centers were selected based on their high pediatric epilepsy caseload and accessibility for recruitment purposes.

Participants and sampling criteria

The inclusion criteria were as follows: 1. Diagnosis of epilepsy is confirmed through clinical evaluation and review of medical records by a pediatric neurologist, with supporting electroencephalography (EEG) findings where available, in accordance with the International League Against Epilepsy (ILAE) diagnostic criteria [6]; 2. Age between three and 12 years; 3. A primary caregiver (parent) who is willing and able to complete the questionnaire is also needed.

The exclusion criteria were as follows: 1. Presence of severe intellectual disability, cerebral palsy, or visual impairment; 2. Comorbid chronic or critical illness; 3. The caregiver is unable to understand the questionnaire or is unwilling to participate.

A total of 226 questionnaires [7] were distributed, and 200 valid responses were analyzed after excluding incomplete or inconsistent data. Given the exploratory nature of this cross-sectional study and the lack of prior prevalence data on screen exposure patterns among children with epilepsy in Egypt, the sample size was estimated using convenience sampling based on expected recruitment rates at the two participating hospitals over 12 months. A target of 200 participants was deemed sufficient to detect moderate effect sizes in multivariate logistic regression models with acceptable power (>80%), assuming an alpha level of 0.05.

Research tools

General Information Questionnaire

This instrument collected data across four domains: A. Child demographics: age, sex, and sibling status; B. Clinical characteristics: family history of epilepsy, seizure frequency (≤10 vs. >10 lifetime episodes), average seizure duration (<1 minute, 1-5 minutes, or >5 minutes), and antiepileptic drug regimen (monotherapy vs. polytherapy); C. Caregiver/family factors: urban/rural residence, parental education level (≤high school vs. college/university), occupation, and family income; D. Parental screen regulation behaviors: five items assessing brightness adjustment reminders, caregiver discussion of screen content, and device access restrictions [7,8].

Caregiver Discussion on Screen Content

This variable assessed whether caregivers engaged in discussions with their children about the content viewed on screens. It was evaluated using a single item from the parental screen regulation behaviors section of the General Information Questionnaire: "Do you discuss with your child what they watch on screens?" Responses were dichotomized as yes or no. This item was adapted from validated instruments used in pediatric media research [7, 8] and reflects a component of active parental mediation known to influence children's screen behaviors. While this approach does not capture the frequency or quality of such discussions, it serves as a practical indicator of caregiver involvement in digital content supervision.

Self-compiled screen exposure questionnaire

This tool was developed based on prior literature and pilot testing [7-9] and evaluated. This was as follows: Screen time: median daily exposure on school days and weekends (minutes/day), stratified by device type (mobile phones, tablets, televisions, computers); Screen activities: predominant use categories (cartoons, short videos, educational content, games); Seizure-screen association: retrospective reporting of seizure occurrence during device use and concurrent activity type; Definitions: aligned with the 2016 American Academy of Pediatrics (AAP) guidelines, excessive screen time was defined as >1 hour/day on school days or >2 hours/day on weekends [10].

Development and validation

The Self-Compiled Screen Exposure Questionnaire was developed through a systematic review of validated screen time instruments used in pediatric populations [7-9]. Before full-scale data collection, the questionnaire underwent face and content validity assessments by a panel of three pediatric health experts and two behavioral scientists. Minor modifications were made to enhance clarity and cultural appropriateness for Egyptian parents.

A pilot study involving 20 caregivers was conducted to assess feasibility, comprehension, and internal consistency. The Cronbach's alpha coefficient for the screen exposure scale was 0.81, indicating acceptable reliability.

Data collection procedures

Data collection was conducted by four trained research assistants who had completed a one-week training program covering ethical guidelines, questionnaire administration techniques, and basic epilepsy awareness. All interviews were conducted in Arabic, the native language of the participants, in private rooms within outpatient clinics to ensure confidentiality.

To ensure data accuracy, a random sample of 10% of completed questionnaires was re-entered by a second researcher to verify consistency. Any discrepancies were resolved through consensus discussion.

Statistical analysis

Data were analyzed using IBS SPSS Statistics software, version 17.0 (IBM Corp., Armonk, NY). Continuous variables with normal distribution were expressed as mean ± standard deviation (xˉ±s); non-normally distributed data were reported as median (interquartile range). Categorical variables were summarized as frequencies and percentages. The Wilcoxon rank-sum test (for binary comparisons) or the Kruskal-Wallis H test (for multi-category comparisons) was used to assess associations between sociodemographic/clinical factors and screen time. Multivariate logistic regression was used for all pre-selected independent variables that were included in the model using a backward elimination approach (removal threshold: p > 0.20) to identify predictors of excessive screen exposure. The resulting adjusted odds ratios (AOR) represent the independent contribution of each variable after controlling for others in the model. Statistical significance: A p-value < 0.05 was considered statistically significant.

## Results

Demographic and clinical characteristics

A total of 200 children with epilepsy were included in the study. Table 1 presents the demographic and clinical characteristics of the participants, including screen device ownership and pre-bedtime screen use patterns.

**Table 1:** Demographic and clinical characteristics, screen device ownership, and pre-bedtime screen use in the studied group

Variable	Number of cases (%)
Age group	
3–≤6 years (preschool)	80 (40%)
6–12 years (school age)	120 (60%)
Sex	
Male	101 (50.5%)
Female	99 (49.5%)
Total number of epileptic seizures	
≤10	184 (92%)
>10	16 (8%)
Medication regimen	
Monotherapy (1 drug)	114 (57%)
Polytherapy (2 drugs)	68 (34%)
Polytherapy (≥3 drugs)	18 (9%)
Family residence	
Urban	118 (59%)
Rural	82 (41%)
Parents' marital status	
Married	187 (93.5%)
Other (e.g., divorced, widowed)	13 (6.5%)
Parents education level	
University degree or higher	87 (43.5%)
Secondary school	76 (38%)
Preparatory school or below	37 (18.5%)
The child has a screen device	
Yes	39 (19.5%)
No	161 (80.5%)
Screen use before bedtime	
Yes	78 (39%)
No	122 (61%)

Screen exposure by age and sex

As shown in Table 2, screen exposure varied significantly by age and sex. Children aged between three and six years spent significantly more time on screens compared to those aged between six and 12 years, both on school days (72 ± 16 minutes vs. 62 ± 14 minutes; p < 0.001) and weekends (134 ± 34 minutes vs. 123 ± 29 minutes; p < 0.001), resulting in higher mean daily screen time (98 ± 25 minutes vs. 88 ± 27 minutes; p < 0.001). Male children also had higher screen time than female children on both school days (74 ± 13 minutes vs. 65 ± 10 minutes; p < 0.01) and weekends (131 ± 27 minutes vs. 122 ± 30 minutes; p < 0.01), leading to a greater overall daily screen exposure (97 ± 22 minutes vs. 91 ± 19 minutes; p < 0.01).

**Table 2:** Screen exposure in children with epilepsy SD: standard deviation

Variable	Number of cases	Screen time on school days (mean ± SD)	Screen time on weekends (mean ± SD)	Mean daily screen time (mean ± SD)
Age (in years)				
3-≤6	80	72 ± 16	134 ± 34	98 ± 25
6-12	120	62 ± 14	123 ± 29	88 ± 27
P-value (Wilcoxon rank-sum test)	--	<0.001	<0.001	<0.001
Sex				
Male	101	74 ± 13	131 ± 27	97 ± 22
Female	99	65 ± 10	122 ± 30	91 ± 19
P-value (Kruskal-Wallis H test)	--	<0.01	<0.01	<0.01

Univariate analysis

Univariate analysis (Table 3) revealed that age group and sex were significantly associated with increased screen time (p < 0.01 for both). However, seizure frequency (p = 0.23) and medication regimen (p = 0.18) did not show statistically significant associations. Parental education level was significantly related to screen exposure (p = 0.02), with lower parental education correlating with higher screen time. Family residence (urban vs. rural) showed no significant association (p = 0.11).

**Table 3:** Univariate analysis of results *: significant

Variable	Comparison type	Statistical test used	P-value
Age group (3–≤6 years vs. 6–12 years)	Binary	Wilcoxon rank-sum test	<0.001*
Sex (male vs. female)	Binary	Wilcoxon rank-sum test	<0.01*
Seizure Frequency (≤10 episodes vs. >10 episodes)	Binary	Wilcoxon rank-sum test	0.23
Medication regimen (monotherapy vs. polytherapy)	Multi-category	Kruskal-Wallis H test	0.18
Parental education level (university degree or higher vs. secondary school vs. preparatory school or below)	Multi-category	Kruskal-Wallis H test	0.02*
Family residence (urban vs. rural)	Binary	Wilcoxon rank-sum test	0.11

Bivariate analysis

Bivariate logistic regression analysis (Table 4) identified several significant predictors of excessive screen use. Children aged between three and six years were 1.67 times more likely to engage in excessive screen use than older children (odds ratio (OR): 1.67; 95% CI: 1.11-2.51; p = 0.01). Male children had nearly double the odds of excessive screen use compared to females (OR: 1.92; 95% CI: 1.29-2.86; p = 0.001). Longer seizure duration (>5 minutes) was also significantly associated with increased risk (OR: 2.44; 95% CI: 1.37-4.34; p = 0.002). Lower parental education level (≤high school) was strongly linked to excessive screen use (OR: 3.29; 95% CI: 1.98-5.46; p < 0.001). Finally, pre-sleep screen use was a major predictor, increasing the odds of excessive screen time by more than threefold (OR: 3.39; 95% CI: 2.04-5.64; p < 0.001).

**Table 4:** Bivariate analysis of results *: significant; Pre-sleep screen use refers to the use of electronic screen-based devices within one hour before bedtime.

Variable	Outcome: excessive screen use (>1 hour/day on school days or >2 hours/day on weekends)	Odds ratio (OR)	95% CI (confidence interval)	P-value
Age group (3–≤6 years vs. 6–12 years)		1.67	1.11–2.51	0.01*
Sex (male vs. female)		1.92	1.29–2.86	0.001*
Seizure duration (>5 minutes vs. ≤5 minutes)		2.44	1.37–4.34	0.002*
Parental education level (≤high school vs. college/university)		3.29	1.98–5.46	<0.001*
Pre-sleep screen use (yes vs. no)		3.39	2.04–5.64	<0.001*

Multivariate logistic regression analysis

Multivariate logistic regression (Table 5) confirmed that pre-sleep screen use (adjusted odds ratio (AOR): 3.41; 95% CI: 1.89-6.16; p < 0.001) and low parental education level (AOR: 3.39; 95% CI: 1.98-5.82; p < 0.001) remained the strongest independent predictors of excessive screen use. Additionally, caregiver discussion of screen content was found to be a significant factor, with a lack of discussion increasing the likelihood of excessive screen use by over threefold (AOR: 3.11; 95% CI: 1.44-6.73; p = 0.004). Seizure duration >5 minutes also independently predicted excessive screen exposure (AOR: 3.07; 95% CI: 1.62-5.81; p = 0.001). Notably, variables such as family income, device ownership, and total seizure count did not reach statistical significance (p > 0.05).

**Table 5:** Multivariate logistic regression analysis of results *: significant; The AOR indicates how strongly a factor is associated with the outcome (in this case, excessive screen time) after accounting for other variables in the model. An AOR greater than one means the factor increases the likelihood of the outcome.

Variable	Predictor of excessive screen use (>1 hour/day on school days or >2 hours/day on weekends)	Adjusted odds ratio (AOR)	95% CI (confidence interval)	P-value
Pre-sleep screen use (yes vs. no)		3.41	1.89 – 6.16	<0.001*
Parental education level (≤high school vs. college/university)		3.39	1.98–5.82	<0.001*
Caregiver discussion on screen content (yes vs. no)		3.11	1.44–6.73	0.004*
Seizure duration (>5 minutes vs. ≤5 minutes)		3.07	1.62–5.81	0.001*

## Discussion

In the last two decades, excessive screen time has emerged as a significant public health issue in pediatric age groups, especially in children with neurological disorders like epilepsy. This study identified that children aged between three and six years with epilepsy had a mean daily screen exposure time of 98 ± 25 minutes, exceeding the AAP guidelines of ≤60 minutes per day for preschoolers. Similarly, children aged between six and 12 surpass the AAP recommendation per day for school-aged children [10]. Collectively, these results demonstrate that the average daily screen time of all participants was 92 minutes, which is a considerable deviation from evidence-based guidelines and acts to highlight the pressing necessity for targeted interventions for this at-risk population. The percentage of children with epilepsy participating in this research who had high levels of screen use is aligned with global trends found within neurotypical populations. For instance, Madigan et al. [1] reported that 70% of Canadian preschoolers exceeded screen time guidelines, while Ren and Zhu [8] reported comparable rates (75%) for Chinese schoolchildren. However, the screen exposure durations in this cohort were higher than those documented in Western studies involving children with epilepsy. Çelen Yoldaş et al. [11] observed mean daily screen times of 150 minutes in Turkish children with epilepsy, potentially attributable to cultural differences in parental regulation practices. Egyptian caregivers in this study may impose stricter restrictions, as evidenced by the prevalence of a 19.5% device ownership rate and 39% pre-sleep screen use. Prior research has associated such restrictive strategies with reduced screen exposure [1]. Nevertheless, the high rates of excessive participant screen time point to deficits in guideline adherence, where more parental education and policy implementation are necessary.

In this study, of the 78 patients (39%) using screens before bedtime, retrospective data indicated that 12 (6%) had experienced seizures during screen use. While no statistically significant difference in screen time was observed between children with and without seizure histories, this null result may stem from post hoc behavioral modifications. Parents of children who have previously had experience with seizure-screen associations may have had more restrictions imposed, thereby concealing possible associations. Furthermore, the small sample size (n=200) may have had insufficient statistical power to identify associations. These findings support Gregory et al. [12], who noted that photosensitive seizures, triggered by flickering visuals, high-contrast imagery, or rapid scene transitions, occur in 10% of individuals with epilepsy, compared to <0.5% in the general population. Although this study did not systematically assess photosensitivity, future investigations should employ electroencephalographic testing to evaluate its role in screen-related seizure triggers. Longitudinal designs with larger cohorts and objective screen monitoring tools (e.g., wearable devices) are warranted to elucidate causal relationships between screen exposure and seizure frequency.

This study further revealed that pre-sleep screen use significantly increased the risk of excessive screen exposure, with an AOR of 3.41 (95% CI: 1.89-6.16, p<0.001). Such findings align with experimental evidence demonstrating that evening screen exposure suppresses melatonin secretion, delays sleep onset, and disrupts circadian rhythms [2]. These effects are particularly concerning for children with epilepsy, as sleep deprivation is a well-documented seizure precipitant [5]. Caregivers permitting pre-sleep screen use may inadvertently adopt an indulgent digital parenting style characterized by lax enforcement of media boundaries. Indulgent parenting correlates with higher screen time in neurotypical children [9], and this study extends those observations to children with epilepsy. Clinically, these results emphasize the need to include sleep hygiene education in epilepsy management routines, with a particular emphasis on pre-sleep screen time cessation to minimize excessive exposure and seizure vulnerability.

Parental education emerged as a pivotal sociodemographic determinant with an AOR of 3.39 (95% CI: 1.98-5.82, p<0.001). These results align with international studies demonstrating inverse relationships between the education of caregivers and screen time for children. Highly educated parents employ more structured regulation approaches, such as a time limit and co-viewing, whereas less-educated caregivers utilize screens as a means of supervision [13]. Future research should disentangle the relative influence of maternal versus paternal educational levels and digital supervision patterns. Another study [14] speculated that greater maternal involvement in everyday childcare might increase their influence on weekend screen use. Future interventions should prioritize low-literacy populations, offering culturally adapted resources to enhance parental awareness of screen-related risks and evidence-based regulation techniques.

The study also identified prolonged seizure duration (>5 minutes) as a predictor of weekend overuse (OR: 3.07; 95% CI: 1.62-5.81). This association could reflect postictal fatigue, caregiver anxiety about outdoor activities, or heightened reliance on screens for entertainment. Prolonged seizure activity often necessitates recovery periods, during which guardians may prioritize safety over physical engagement, thus inadvertently reinforcing sedentary habits. Additionally, weekend schedules may afford greater unstructured time, amplifying disparities between children with brief versus extended seizure histories. These results extend prior research on epilepsy-related activity restrictions by highlighting the interplay between clinical severity and digital behavior. Clinically, this underscores the need for multidisciplinary healthcare teams to address both seizure management and lifestyle changes, promoting alternative activities such as supervised recreation or adapted sports. Collectively, these results support a multi-faceted reduction of screen time among children with epilepsy. Key targets for intervention include imposing pre-bedtime screen bans, enhancing parent education initiatives, and involving disease-specific variables like seizure duration. Healthcare providers should include digital hygiene counseling within routine epilepsy care, capitalizing on teachable moments during clinic visits [5]. Additionally, culturally appropriate educational materials, particularly for low-literacy caregivers, are necessary to close knowledge gaps. Longitudinal research examining associations between screen exposure and seizure dynamics is needed, with objective outcomes (e.g., actigraphy, screen use diaries) to validate self-reported information. Comparative studies across diverse geographic and socioeconomic contexts are also required to address global disparities in digital parenting practices [5]. Nursing healthcare professionals play an important role in addressing excessive screen exposure among children with epilepsy by integrating digital hygiene education into routine care. They can assess screen time habits, provide evidence-based guidance on age-appropriate limits, and counsel families on the risks associated with prolonged exposure, particularly before bedtime. They can also support caregivers in implementing structured screen use regulations and promoting healthier alternatives, such as supervised physical activities and interactive play. In addition, nurses can tailor educational interventions to address sociodemographic factors, such as parental education levels, to ensure that families from diverse backgrounds receive the necessary tools and knowledge to manage screen exposure effectively.

Limitations and future work

The study's cross-sectional design limits the causal relationship between screen exposure and seizure outcomes, necessitating longitudinal cohort studies to determine bidirectional screen time effects on seizure frequency and vice versa. The study relied on self-reported data from caregivers, which may be subject to recall bias, particularly among families with lower literacy levels. Screen time patterns, pre-sleep use, and even seizure-related events were reported retrospectively, which may lead to inaccuracies in frequency or duration estimates. In the future, objective monitoring of screen use by using wearable devices can be employed to validate self-reports and examine real-time associations between seizures and screen use. Convenience sampling restricted findings to urban Egyptian populations, highlighting the need for cultural comparisons across low- and middle-income countries to address global disparities in digital parenting practices. Photosensitivity status, a critical seizure trigger, was not systematically assessed, underscoring the importance of integrating electroencephalographic testing in future intervention trials. Such trials should also test targeted strategies to reduce screen time, including gamified parental apps or telehealth counseling, particularly for families with low health literacy. Addressing these gaps will improve knowledge of screen dangers in children with epilepsy and guide the development of culturally sensitive, evidence-based interventions.

## Conclusions

In conclusion, this study delineates modifiable risk factors for excessive screen exposure in children with epilepsy, offering actionable insights for clinicians, educators, and policymakers. By addressing parental regulation behaviors, seizure-related sedentary tendencies, and educational disparities, stakeholders can mitigate screen-related harm while promoting holistic development in this high-risk population.
